# Binding of respiratory syncytial virus particles to platelets does not result in their degranulation *in vitro*

**DOI:** 10.1099/acmi.0.000481.v3

**Published:** 2023-07-13

**Authors:** Anke J. Lakerveld, Elisabeth A. van Erp, Puck B. van Kasteren

**Affiliations:** ^1^​ Centre for Infectious Disease Control, National Institute for Public Health and the Environment (RIVM), Bilthoven, The Netherlands

**Keywords:** P-selectin expression, platelets, RSV, respiratory syncytial virus

## Abstract

Respiratory syncytial virus (RSV) is a major cause of severe respiratory infection in infants and the elderly. The mechanisms behind severe RSV disease are incompletely understood, but a dysregulated immune response probably plays an important role. Platelets are increasingly being recognized as immune cells and are involved in the pathology of several viruses. The release of chemokines from platelets upon activation may attract, for example, neutrophils to the site of infection, which is a hallmark of RSV pathology. In addition, since RSV infections are sometimes associated with cardiovascular events and platelets express several known RSV receptors, we investigated the effect of RSV exposure on platelet degranulation. Washed human platelets were incubated with sucrose-purified RSV particles. P-selectin and CD63 surface expression and CCL5 secretion were measured to assess platelet degranulation. We found that platelets bind and internalize RSV particles, but this does not result in degranulation. Our results suggest that platelets do not play a direct role in RSV pathology by releasing chemokines to attract inflammatory cells.

## Data Summary

The data supporting the findings of this study are available within the article.

## Full-text

Respiratory syncytial virus (RSV) is a major cause of severe respiratory infections, especially in infants and the elderly. Most infants experience only mild upper respiratory tract infections, but RSV infections can also develop into severe lower respiratory tract infections requiring hospitalization [1, 2]. There is no specific treatment or vaccine available for RSV, except for monoclonal antibody prophylaxis by palivizumab and its recent successor nirsevimab [3, 4]. The exact mechanisms underlying severe RSV disease are currently unknown, but a dysregulated immune reaction probably contributes to lung damage (reviewed in [5]). Excessive infiltration of immune cells into the lungs is often seen in severe RSV disease [6, 7]. In order to develop novel treatment options and vaccines, a better understanding of RSV immunopathology is necessary.

Various types of immune cells may be involved in immunopathology. Although platelets are primarily known for their function in thrombosis and haemostasis, they have recently received more attention for their immunological role. A broad range of pattern-recognition receptors (PRRs) are expressed on platelets, including toll-like receptors (TLRs), C-type lectin receptors (CLRs) and NOD-like receptors (reviewed in [8]). Upon activation, platelets release the contents of α-granules and dense granules containing immunomodulatory cytokines, chemokines and growth factors. These molecules can modulate the immune response indirectly by attracting immune cells to the site of infection, thereby contributing to cellular infiltration. In addition, when platelets become activated, P-selectin, normally residing on the membrane of cytosolic α-granules, becomes exposed on the platelet surface. Platelets can directly interact with other immune cells via binding of P-selectin to its receptors present on neutrophils, monocytes and macrophages (reviewed in [8]).

The immunomodulatory role of platelets has already been shown for several viral infections, such as dengue virus, human immunodeficiency virus (HIV) and influenza virus [9–11]. Platelet hyperactivity has also been implicated in the severe pathology of severe acute respiratory syndrome coronavirus-2 (SARS-CoV-2) infections [12, 13]. There are several indications suggesting that platelets may also play a role in RSV infections. Interestingly, increased expression of genes associated with platelets was found in RSV lower respiratory tract infections in infants [14]. Cardiovascular complications, to which platelets may contribute, have also been associated with RSV infections (reviewed in [15]). Furthermore, multiple receptors known to bind RSV are expressed on the platelet membrane, such as TLR4, CX3CR1 and DC-SIGN [16–18]. Additionally, platelet CLRs potentially bind glycans present on the highly glycosylated RSV surface proteins [19]. While platelets are normally not present in the healthy lung lumen, RSV infection probably leads to epithelial damage enabling various immune cells, including platelets, to enter the alveolar space where they will encounter RSV particles. Likewise, platelets have been found in the lungs of allergen-challenged mice and in mice with LPS-induced inflammation [20, 21]. Furthermore, lungs have been found to harbour platelet precursor cells (megakaryocytes), suggesting that some platelet production occurs in this tissue in addition to the bone marrow as a main production site [22]. In this study, we examined whether platelets may be involved in RSV disease, specifically whether exposure to RSV particles leads to platelet degranulation *in vitro*.

Washed human platelets were isolated from 3.2 % sodium citrate anticoagulated whole blood from healthy adult volunteers as described previously [23]. People using aspirin within 2 weeks prior to blood drawing were excluded. Age and sex were not disclosed and deemed irrelevant for this study, although it is noted that a difference in platelet activation between boys and girls during RSV-induced bronchiolitis has been described previously [24]. Briefly, whole blood was centrifuged at 160 ***g*** for 15 min without a brake to obtain platelet-rich plasma (PRP). PRP was further centrifuged in the presence of anticoagulants to ultimately obtain washed platelets, which were resuspended in HEPES Tyrode buffer at a concentration of 100×10^6^ platelets ml^–1^. Platelets were rested for 30 min before continuing with experiments.

Human RSV-A2 (ATCC VR-1540), RSV-98-25147 X (RSV-A strain from the Netherlands, 1998, here referred to as RSV-X; GenBank FJ944820.1) and recombinant RSV-X-GFP [25] were propagated in HEp-2 cells (ATCC Cat. no. CCL-23, RRID:CVCL_1906). Virus stocks were purified between layers of 10 and 50 % sucrose by ultracentrifugation and subsequently snap-frozen. Virus stored at −80 °C was rapidly thawed at 37 °C shortly before use. The 50 % tissue culture infective dose (TCID_50_) per millilitre was determined on Vero cells (ATCC Cat. no. CCL-81, RRID:CVCL_0059) using the Spearman and Karber method [26] and converted to plaque-forming units (PFU) per millilitre by multiplying by 0.69.

**Fig. 1.**
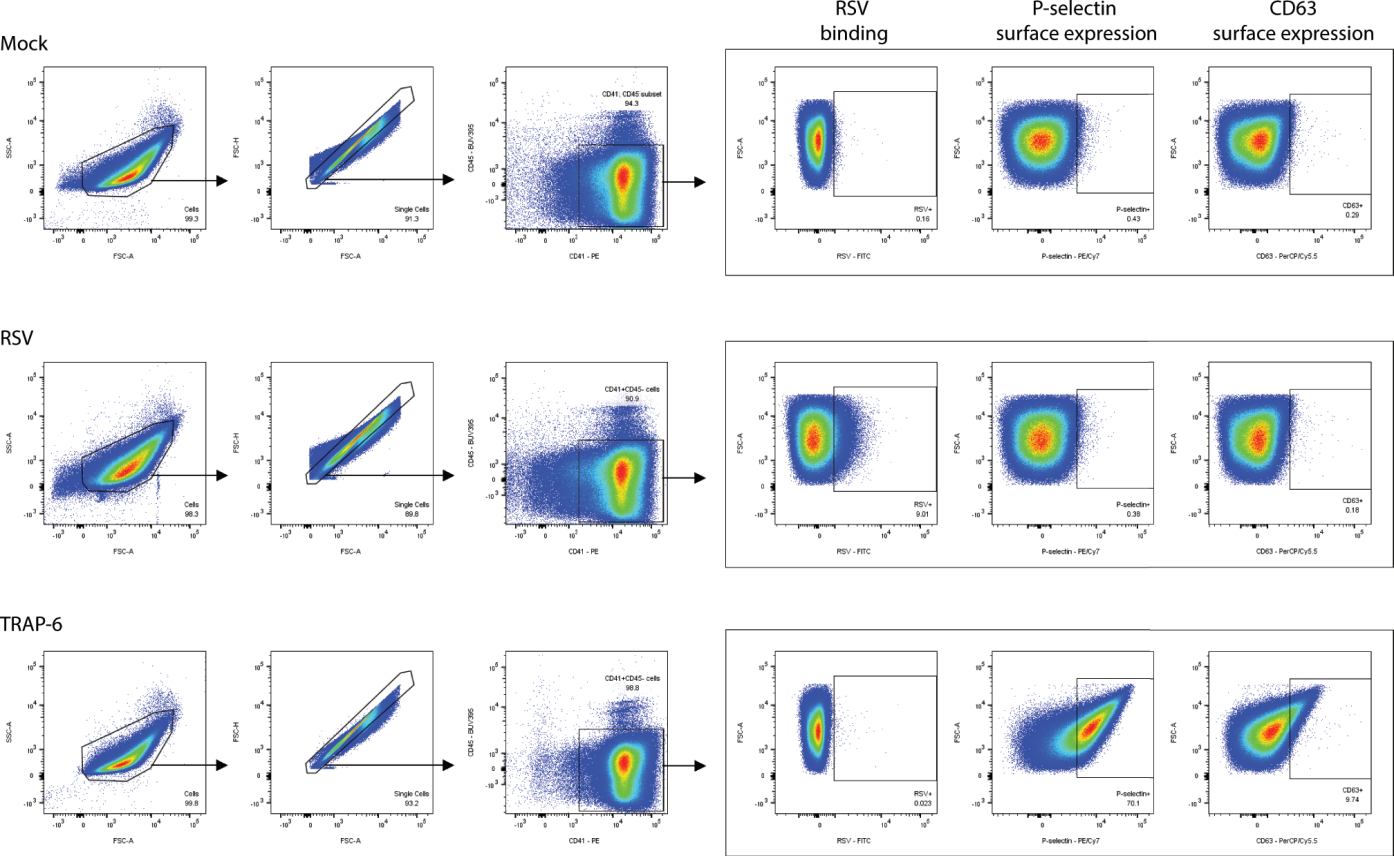
Gating strategy to assess RSV binding (FITC-conjugated 131-2G) and P-selectin (CD62P, PE/Cy7-conjugated AK4) and CD63 (PerCP/Cy5.5-conjugated H5C6) surface expression on single CD41+ (PE-conjugated HIP8) CD45– (BUV395-conjugated HI30) platelets after mock, RSV or TRAP-6 incubation. Flow cytometry plots are shown for one representative donor and made using FlowJo software (RRID:SCR_008520).

**Fig. 2.**
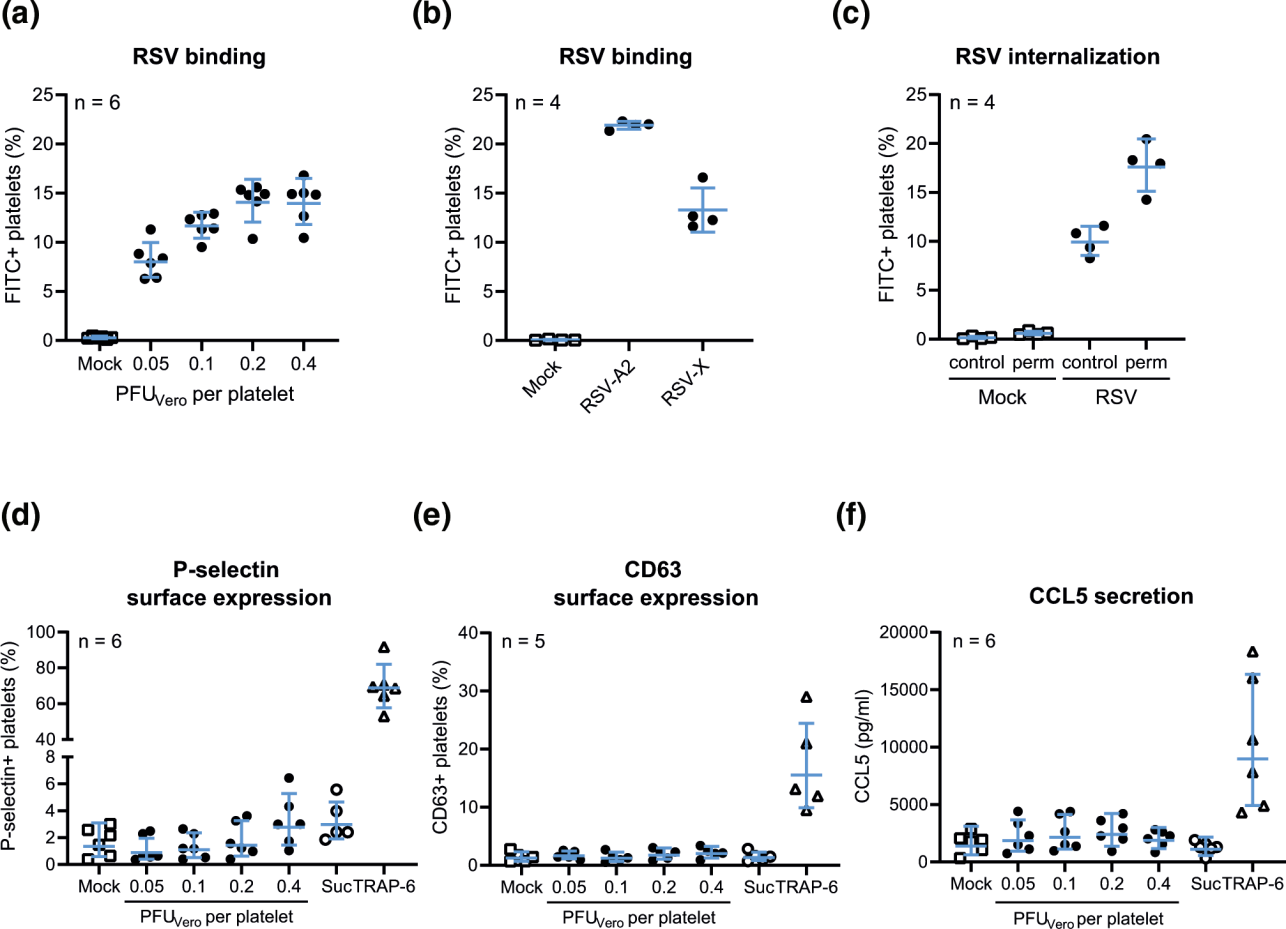
Platelets bind and internalize RSV particles, which does not lead to platelet degranulation *in vitro*. (**a**) Washed platelets were incubated for 1 h with sucrose-purified RSV-A2 at the indicated concentrations and analysed by flow cytometry after staining with an FITC-conjugated antibody specific for the RSV attachment protein G (131-2G). Graphs show the percentage of FITC-positive platelets of different donors (*n*=4–6). (**b**) Washed platelets were incubated for 1 h with 0.1 PFU_Vero_ per platelet of RSV strains A2 (ATCC VR-1540) or X (GenBank FJ944820.1) and analysed as in (**a**). (**c**) Following 1 h of incubation with RSV-A2 at 0.1 PFU_Vero_ per platelet, platelets were fixed and permeabilized with a cytofix/cytoperm kit, stained as in (**a**) and measured with flow cytometry to assess RSV internalization. Following 1 h of incubation with RSV-A2 at 0.1 PFU_Vero_ per platelet, platelet degranulation was assessed by measuring P-selectin (**d**) and CD63 (**e**) surface expression and CCL5 secretion (**f**). Each data point indicates the average of technical duplicates from a single platelet donor. Abbreviations: perm, permeabilized; PFU, plaque-forming units; suc, sucrose control; TRAP-6, thrombin receptor activating peptide-6.

To assess RSV binding, washed platelets were incubated for 1 h at 37 °C with sucrose-purified RSV-A2 diluted in HEPES Tyrode buffer in different concentrations, indicated as plaque-forming units determined on Vero cells (PFU_Vero_) per platelet. As a mock control, only HEPES Tyrode buffer was added to the platelets. Following incubation, platelets were stained with an FITC-labelled antibody specific for the RSV attachment protein G (Merck Millipore Cat. no. MAB858-2F-5, clone 131-2G) and cell surface markers anti-human CD41-PE (BioLegend Cat. no. 303706, RRID:AB_314376, clone HIP8) and anti-human CD45-BUV395 (BD Biosciences Cat. no. 563792, RRID:AB_2869519, clone HI30) and analysed by flow cytometry on an LSR Fortessa X-20 (BD Biosciences). Flow cytometry plots detailing the gating strategy for single CD41+CD45− platelets can be found in Fig. 1. Increasing amounts of RSV resulted in an increasing percentage of FITC-positive platelets up to approximately 15 % (*n*=6), thus indicating platelets with surface-attached RSV particles (Fig. 2a). To investigate whether platelets were able to bind different RSV strains, both RSV-A2 and RSV-X were added to platelets at a ratio of 0.1 PFU_Vero_ per platelet for 1 h (*n*=4). Fig. 2(b) shows that platelets can bind both RSV-A2 and RSV-X.

Platelets are known to exhibit endocytic activity [11, 27]. To determine whether platelets can also internalize RSV particles, platelets were fixed and permeabilized with a cytofix/cytoperm kit (BD Biosciences) following 1 h of incubation and stained for RSV as described above (*n*=4). Fig. 2(c) shows a clear increase in the percentage of platelets positive for RSV when permeabilized compared to the non-permeabilized controls (18 and 10 %, respectively). This indicates that besides binding of RSV to the platelet surface, RSV particles are also localized inside the platelet and are thus internalized.

P-selectin is a well-known platelet activation marker which resides on the membrane of α-granules in resting platelets. After activation and granule release, P-selectin is exposed on the surface of platelets, where it can be measured with flow cytometry using anti-human CD62P-PE/Cy7 (BioLegend Cat. no. 304922, RRID:AB_2572028, clone AK4) to obtain an indication of the extent of platelet degranulation. When exposing platelets to increasing amounts of RSV, only a small fraction of platelets (1–3 %, *n*=6) show surface expression of P-selectin (Fig. 2d). Furthermore, the minor increase that is observed at the highest RSV concentrations appears to be the result of sucrose present in the virus stocks, as it is also observed in the sucrose control condition (‘Suc’ in Fig. 2d) where a similar concentration of sucrose was added as was present at the highest RSV concentration. Using thrombin receptor activating peptide-6 SFLLRN (TRAP-6, BACHEM Cat. no. H-8365) at a concentration of 25 µM as a positive control, we show that the platelets used in our experiments are indeed able to detectably express P-selectin on their surface, as approximately 70 % of platelets stain positive for P-selectin after TRAP-6 incubation. Surface expression of CD63, present on the membrane of dense granules in resting platelets, is another well-known platelet degranulation marker. Similar to P-selectin, we found that CD63 [stained with anti-human CD63-PerCP/Cy5.5 (BioLegend Cat. no. 353020, RRID:AB_2561685, clone H5C6)] is not expressed on RSV-exposed platelets (*n*=5) while it is expressed on TRAP-6-stimulated platelets (Fig. 2e). Together, these results suggest that RSV particles do not induce degranulation of platelets.

Platelet degranulation results in the release of various molecules including cytokines, chemokines and growth factors. As an example, Fig. 2(f) shows that secretion of the platelet-associated chemokine CCL5 (determined using a custom LegendPlex assay; Biolegend) is not increased following a 1 h incubation of platelets with RSV particles, while CCL5 secretion does show an increase upon TRAP-6 stimulation (*n*=6). This finding is in line with the absence of an effect of RSV incubation on P-selectin and CD63 surface expression and supports the notion that RSV exposure does not result in platelet degranulation. Strikingly, we did observe increased concentrations of several other (platelet-related) growth factors and chemokines (e.g. VEGF and CCL2) in the platelet supernatant following incubation with RSV. However, upon further inspection, these molecules turned out to originate from the virus stocks and were thus in fact not platelet-derived. Interestingly, others have previously also shown co-purification of host proteins with RSV, speculating that these were either virion-associated or carried within extracellular vesicles [28, 29]. These findings highlight the importance of including proper controls when working with cell-culture-derived materials such as virus stocks.

**Fig. 3.**
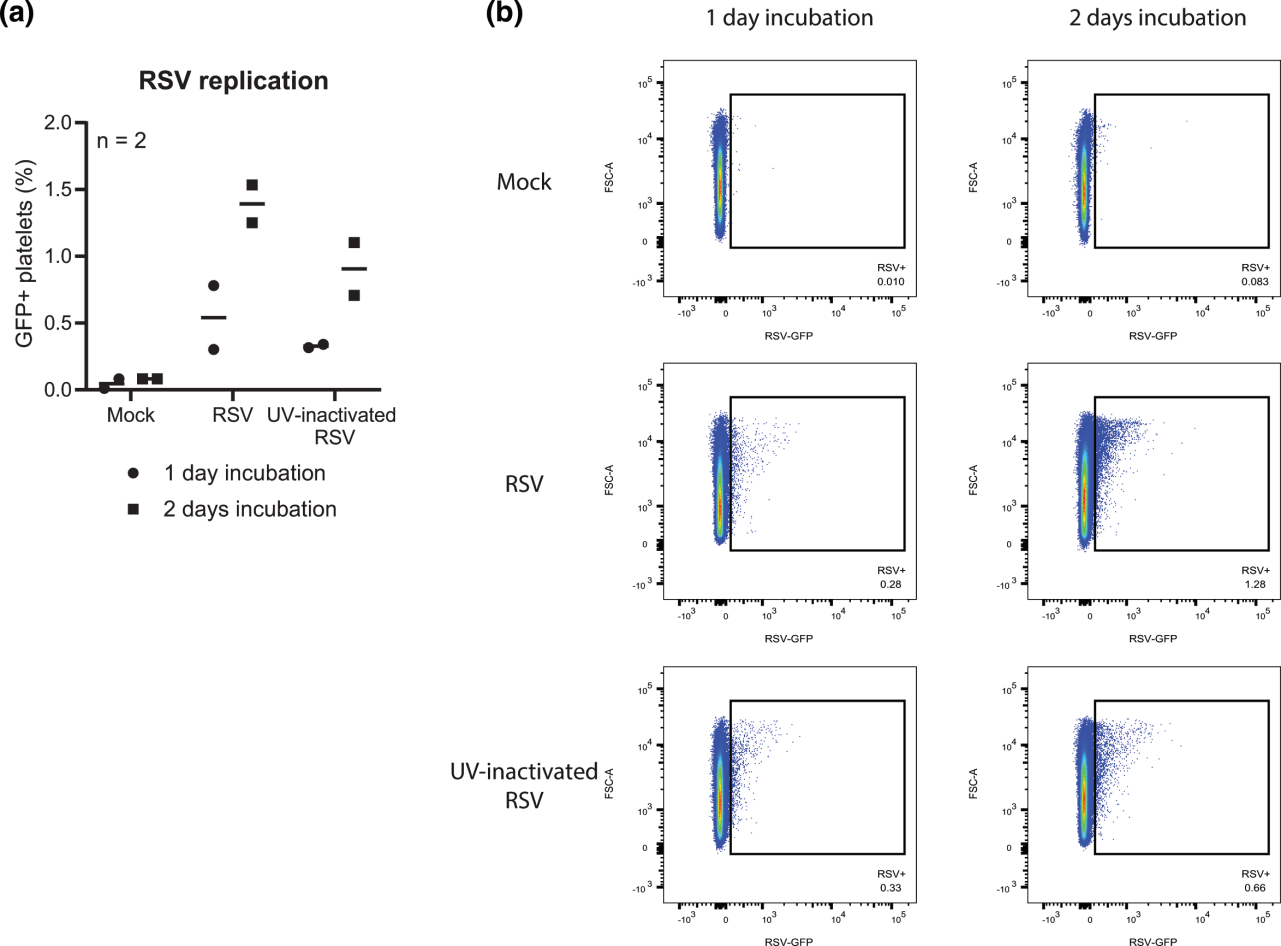
RSV replication in platelets is limited. CD41+CD45− platelets were gated as shown in Fig. 1. Untreated or UV-inactivated RSV-GFP was added to washed platelets from healthy donors (*n*=2) at a concentration of 0.1 PFU_Vero_ per platelet and incubated for up to 2 days. (**a**) Graph showing the percentage of GFP-positive platelets, potentially indicating expression of virus-encoded genes and thus the initiation of viral replication. Each data point indicates the average of technical duplicates from a single platelet donor. (**b**) Flow cytometry plots for one representative donor made using FlowJo software.

Since we observed internalization of RSV particles by platelets, we also examined whether RSV was able to replicate in platelets, as has been shown before for dengue virus [30]. To this end, we added (untreated or UV-inactivated) recombinant RSV encoding green fluorescent protein (GFP) to platelets (*n*=2). The GFP signal becomes visible when transcription and translation of viral mRNA occurs (which should no longer occur following UV inactivation) and is thereby indicative of the initiation of the viral replication cycle. Up to 2 days after the addition of RSV, the percentage of GFP-positive platelets (as measured with flow cytometry) increased to only 1.5 % in the untreated RSV condition while the UV-inactivated RSV condition had already reached approximately 1.0 % (Fig. 3). This finding suggests strongly that most of the observed GFP signal was due to background, for example uptake of GFP-containing material from the virus stock. RSV replication in platelets therefore appears to be extremely limited and – even if it were to result in infectious progeny – is probably biologically irrelevant.

With this study, we show that platelets are able to bind and internalize RSV particles, but do not release their granules after exposure to RSV *in vitro* and consequently do not appear to secrete chemokines to attract immune cells to the site of infection. Furthermore, very limited replication of internalized RSV could be observed in platelets *in vitro*, which is probably biologically irrelevant. In summary, the observed lack of degranulation and very limited viral replication suggests that a harmful role for platelets in RSV pathology is improbable. In contrast, it is conceivable that RSV in fact suppresses platelet activation, but further experiments are necessary.

The mechanism behind the interaction between RSV and platelets remains unclear. As mentioned previously, platelets contain several receptors known to be able to bind RSV (TLR4, CX3CR1 or DC-SIGN) [16–18]. RSV might bind to platelets through these receptors, but a less specific interaction in which the glycans of RSV surface proteins bind to CLRs on platelets is also possible. Additionally, the RSV attachment protein contains a heparin binding domain that can bind to heparan sulphate proteoglycans [31, 32]. This is also a potential mode of interaction, as has been shown for dengue virus binding to platelets [30].

In contrast to the study of Kullaya *et al.,* in which RSV was shown to induce P-selectin expression on platelets [33], we did not find an induction of P-selectin surface expression after RSV exposure. This discrepancy may be explained by the fact that we exposed the platelets to a much lower concentration of RSV particles, which we believe is physiologically more relevant. With these RSV concentrations, we were able to observe a plateau in RSV-positive platelets, while activity markers remained entirely unaffected. This observation suggests strongly that platelet degranulation is not induced by RSV. Furthermore, we show that sucrose has a small influence on P-selectin expression, which might have played a role in the study of Kullaya *et al.* that also used sucrose-purified RSV. Although all our experiments were performed *in vitro*, which does not completely mimic the *in vivo* situation, the positive control TRAP-6 showed that activation was indeed possible under these *in vitro* conditions.

Whereas platelets may not play a detrimental role in RSV disease severity by attracting immune cells, they might instead exert a protective role by binding and internalizing RSV particles. It has previously been shown that platelets are able to engulf and eliminate pathogens, contributing to pathogen clearance [11, 27, 34]. Indeed, Kullaya *et al.* showed that in the presence of platelets, a lower number of monocytes become infected with RSV, resulting in reduced monocyte activation [33]. Additionally, platelets can also present pathogens to phagocytes and dendritic cells and limit their dissemination into the bloodstream [35, 36]. As RSV is mainly a respiratory pathogen, platelets may present RSV to alveolar macrophages. However, considering the absence of RSV-induced P-selectin surface expression, platelet binding to other immune cells may be limited.

Viral infections are sometimes associated with thrombocytopenia, a decrease in platelet counts [27, 37]. During RSV infection, generally no differences in platelet counts have been noted [38], although sporadically thrombocytosis is observed, an increase in platelet counts [39]. Therefore, binding of RSV particles to platelets does not appear to result in significant changes to platelet counts *in vivo*. Instead of functioning to limit infection throughout the body, some viruses (e.g. HIV) actually use platelets as a vehicle to disseminate through the entire body and promote viral spread [40]. It is highly unlikely that platelets play such a role to any significant extent during RSV infection, as RSV RNA has only rarely been found outside the respiratory tract [41, 42].

In conclusion, based on our findings we consider it unlikely that platelets play a direct role in RSV pathology, especially not a detrimental one. The fact that platelets are able to bind RSV particles might limit viral spread, but at the assessed concentrations platelet degranulation is not induced by RSV and these cells are therefore unlikely to contribute significantly to the excessive cellular infiltration observed during severe RSV disease.
